# Serological evidence for a decline in malaria transmission following major scale-up of control efforts in a setting selected for *Plasmodium vivax* and *Plasmodium falciparum* malaria elimination in Babile district, Oromia, Ethiopia

**DOI:** 10.1093/trstmh/trz005

**Published:** 2019-03-30

**Authors:** Migbaru Keffale, Girma Shumie, Sinknesh Wolde Behaksra, Wakweya Chali, Lotus L van den Hoogen, Elifaged Hailemeskel, Daniel Mekonnen, Menberework Chanyalew, Demekech Damte, Tiruwork Fanta, Temesgen Ashine, Sagni Chali, Kevin K A Tetteh, Dereje Dillu Birhanu, Taye T Balcha, Abraham Aseffa, Chris Drakeley, Tesfaye S Tessema, Haileeyesus Adamu, Teun Bousema, Endalamaw Gadisa, Fitsum G Tadesse

**Affiliations:** 1 Malaria and Neglected Tropical Diseases Directorate, Armauer Hansen Research Institute, POBox 1005, Addis Ababa, Ethiopia; 2 Institute of Biotechnology, Addis Ababa University, POBox 1176, Addis Ababa, Ethiopia; 3 Department of Immunology and Infection, London School of Hygiene and Tropical Medicine, London, UK; 4 Department of Biomedical Sciences, College of Natural and Computational Sciences, Addis Ababa University, POBox 1176, Addis Ababa, Ethiopia; 5 Federal Ministry of Health, Addis Ababa, Ethiopia; 6 Medical Microbiology, Radboud University Medical Center, Nijmegen, The Netherlands

**Keywords:** current infection, malaria elimination, metrics of transmission, serology

## Abstract

**Background:**

Following successful malaria control during the last decade, Ethiopia instituted a stepwise malaria elimination strategy in selected low-transmission areas.

**Methods:**

Cross-sectional surveys were conducted in Babile district, Oromia, Ethiopia from July to November 2017 to evaluate malaria infection status using microscopy and nested polymerase chain reaction (nPCR) and serological markers of exposure targeting *Plasmodium falciparum* and *Plasmodium vivax* apical membrane antigen-1 (AMA-1).

**Results:**

Parasite prevalence was 1.2% (14/1135) and 5.1% (58/1143) for *P. falciparum* and 0.4% (5/1135) and 3.6% (41/1143) for *P. vivax* by microscopy and nPCR, respectively. Antibody prevalence was associated with current infection by nPCR for both *P. falciparum* (p<0.001) and *P. vivax* (p=0.014) and showed an age-dependent increase (p<0.001, for both species). Seroconversion curves indicated a decline in malaria exposure 15 y prior to sampling for *P. falciparum* and 11.5 y prior to sampling for *P. vivax*, broadly following malaria incidence data from district health offices, with higher antibody titres in adults than children for both species.

**Conclusions:**

Malaria transmission declined substantially in the region with continuing heterogeneous but measurable local transmission, arguing in favour of continued and tailored control efforts to accelerate the progress towards elimination efforts.

## Introduction

Ethiopia achieved a remarkable reduction in malaria morbidity and mortality following the major scale-up of control efforts in 2004/2005,^1,2^ in line with global trends during the same period.^3^ Following this success, Ethiopia instituted a stepwise approach to eliminate malaria by 2030 in selected low-transmission areas where control efforts were successful.^4^ With the aim of interrupting local transmission, new tools and strategies were considered, including endorsement of primaquine for transmission blocking in *Plasmodium falciparum* and radical cure for *Plasmodium vivax*. As transmission intensity declines, efforts to find and treat the sparse and heterogeneously distributed remaining infections results in operational challenges,^5^ in part because a large fraction of these infections is present at densities below the detection limit of standard diagnostics.^6^ In order to consolidate gains and progress to elimination, improved tools for infection monitoring are needed.

Serological assays that detect human antibody responses to malaria parasite antigens can be used to characterize current and previous parasite exposure.^7^ Uniquely, serology allows retrospective examination of exposure history, including the effects of interventions and the absence of recent exposure in elimination settings.^8,9^ In the present study, the current and past burden of malaria in Babile, a district nominated for elimination by the Federal Ministry of Health (FMoH) of Ethiopia, is examined by microscopy, molecular assays and serology.

## Materials and methods

### Study area and population

A cross-sectional study was conducted from July to November 2017 in 14 villages that are located at an altitude range of 1280–1450 m above sea level in Babile district within the East Hararghe zone of the Oromia region of Ethiopia. Babile is one of the 239 districts nominated by the FMoH for malaria elimination efforts.^4^ The 14 villages were selected using a computer-randomized list. Within these villages, convenience sampling was undertaken, with participants being invited without any formal random selection. Study approval was obtained from the Ethics Review Committees of the College of Natural Sciences at Addis Ababa University (CNSDO/205/10/2017), Armauer Hansen Research Institute (PO24/17) and Oromia Region Health Bureau (BEFO/AHBIFH/1-8/2700). Informed written consent was obtained from every participant or the parent/guardian of children <18 y of age.

### Sample collection and microscopy

Participants were asked about demographic characteristics, intervention utilization, household characteristics and travel history. Finger prick blood samples were collected from participants to prepare thin and thick blood films and dried blood spots (DBSs) on Whatman 3MM filter paper (Whatman, Maidstone, UK). The DBS samples were air dried and stored individually in sealed plastic bags with self-indicating silica gel beads (Loba Chemie, Mumbai, India; CAS: 112926-00-8) and transported under ambient conditions right after collection from the field site and stored at −20°C until further processing. Giemsa-stained thick blood films were considered negative if no parasites were observed after examining 200 fields.

### Molecular parasite identification and serological assays

A combined approach for serum elution and DNA extraction from 6-mm diameter DBS punches was used.^10^ Proper elution of plasma from DBSs was assessed by the colour change of the spots (to white) as well as the elution (to red/brown) after soaking them 1–2 nights in saponin solution at ambient temperature while on a horizontal shaker. If blood spots did not change colour, still retaining the brownish blood colour, spots were soaked further until a colour change was observed or excluded from analyses as reported before.^11^*Plasmodium* species were identified using nested polymerase chain reaction (nPCR) targeting the small subunit 18S ribosomal RNA gene.^12^ Antibody responses (immunoglobulin G) against apical membrane antigen-1 of *P. falciparum* (AMA-1-3D7) and *P. vivax* (Sal-1) were tested using enzyme-linked immunosorbent assay (ELISA)^9,13^ using blood from malaria-naïve Europeans as negative controls and World Health Organization (WHO) positive control sera (reference, 10/198 and 72/096; National Institute for Biological Standards and Control).^14,15^ The WHO reference positive control sera were included on every plate in six, with fourfold serial dilution starting at the concentration of 1/100 in 0.05% phosphate-buffered saline Tween-20 to translate optical density (OD) values to titres as described before.^16^

### Data analysis

Analyses were performed using the survey command in Stata 13 (StataCorp, College Station, TX, USA) to take into account the sampling approach and in GraphPad Prism 5.0 (GraphPad Software, San Diego, CA, USA). The cut-off for seropositivity among samples was determined as the mean OD of the negative control sera plus 3 standard deviations. Seroconversion curves were modelled using a simple reversible catalytic model fitted by maximum likelihood for all study villages combined.^11^ The log-likelihood of a catalytic conversion model that allowed for a change in transmission occurring at iterative years was plotted to evaluate the time at which seroconversion rates changed for each species.^13^ The time point at which a change in transmission most likely had occurred was determined using maximum log-likelihood. A likelihood ratio test was used to determine if a model with a change in transmission vs one without fitted the data best (i.e., p<0.050). Reverse cumulative distribution plots were used to evaluate differences in the geometric mean antibody titres. Wilcoxon rank-sum test with Bonferroni correction for multiple comparisons was used to test between two continuous variables.

## Results

### Characteristics of study villages and the population

**Figure 1.**
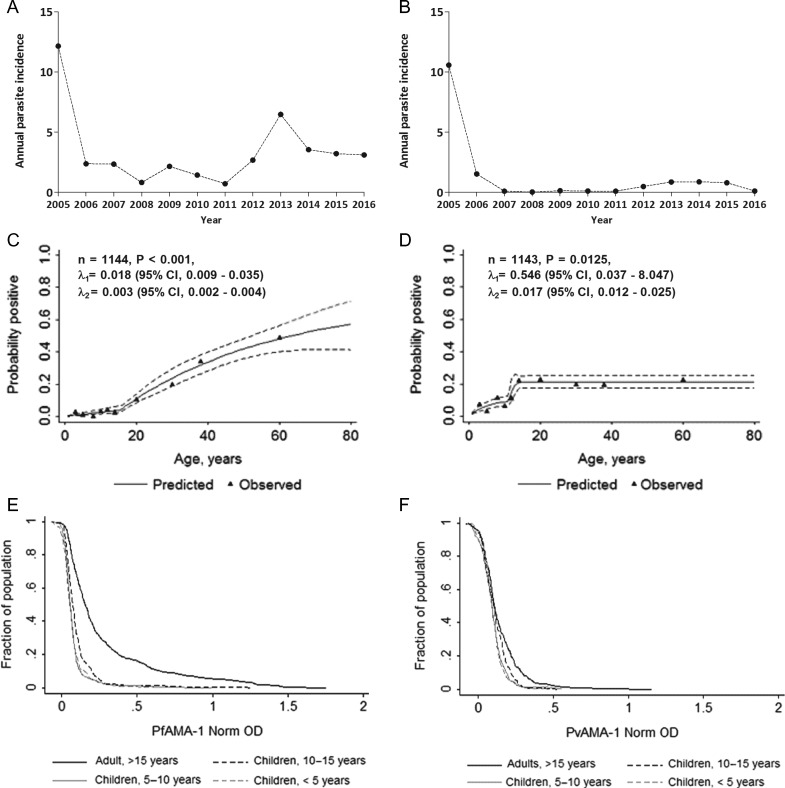
Annual parasite incidence, age seroprevalence plots and trends in antibody titres. Retrospective data from the district health bureau between 2005 and 2016 is presented for (**A**) *P. falciparum* and (**B**) *P. vivax*. Indicated on the *y*-axis is annual parasite incidence per 1000 population with the years indicated on the *x*-axis. Seroconversion curves are presented for (**C**) Pf-AMA (n=1144) and (**D**) Pv-AMA (n=1143) using a simple reversible catalytic model fitted by maximum likelihood. Triangles represent observed data and black lines represent predicted values. Dotted black lines represent upper and lower 95% CIs for the predicted seroprevalence by age. A likelihood ratio test was used to determine if a model with a change in transmission fitted the data best. Associated p-values as well as seroconversion rate estimates pre- and post-change are shown on the plots. Numbers indicate pre-change (λ_1_) and post-change (λ_2_) seroconversion values. Reverse cumulative distribution plots for age groups are indicated for (**E**) Pf-AMA and (**F**) Pv-AMA for the four age groups (adults >15 y of age, black lines; children 10–15 y of age, black dotted lines; children 5–10 y of age, grey lines; children <5 y of age, dotted grey lines). Shown in (E) and (F) are log_10_-transformed normalized OD values on the *x*-axis and the percentage of individuals having the indicated values or higher on the *y*-axis.

A total of 1144 samples, 46–128 per village, were analysed for malaria infection using microscopy and nPCR as well as antibody responses to *P. falciparum* AMA-1 (Pf-AMA) and *P. vivax* AMA-1 (Pv-AMA). The median age of participants was 12 y (interquartile range [IQR] 7–26) and 52.1% (596/1144) of participants were female. None of the participants had measured fever at the moment of sampling. There was substantial variation in long-lasting insecticidal net ownership, ranging from 25.0% to 93.5% between villages (overall 69.0% [789/1144] [95% confidence interval {CI} 55.5–79.9]) (see Supplementary Table 1 for detailed intervention utilization data). A substantial reduction in the incidence and prevalence of malaria has been recorded in the district since 2005, based on malaria control program data from the FMoH and the district health office (Figure 1A, B). Data from the years prior to 2005 could not be retrieved, as structured and organized data recording was only begun in the study districts and across the country following the intensified control measures.

### Malaria infections detected by microscopy, nPCR and seroprevalence

Microscopy detected 1.3% (95% CI 0.5 to 3.3) of *P. falciparum* and 0.4% (95% CI 0.2 to 0.9) of *P. vivax* infections, whereas nPCR detected 5.1% (95% CI 2.5 to 10.1) of *P. falciparum* and 3.6% (95% CI 2.2 to 5.7) of *P. vivax* infections. Antibodies against Pf-AMA were detected in 11.2% (95% CI 6.5 to 18.6) of study participants; for Pv-AMA this prevalence was 13.0% (95% CI 5.3 to 28.6) (Table 1). Parasite prevalence estimates varied between villages (Table 1) (p<0.001) for microscopy (range 0–6.2%), nPCR (range 1.1–24.5%) and antibody responses to Pf-AMA (range 0–38.4%) and Pv-AMA (range 0–75.7%). Of the individuals who were seropositive for Pf-AMA, 17.2% (22/128) were also positive for *P. falciparum* parasites by nPCR compared with 3.5% (36/1016) nPCR positivity among seronegatives (p<0.001). Similarly, *P. vivax* parasite prevalence by nPCR was higher among Pv-AMA seropositive (8.1% [11/149]) compared with seronegative individuals (2.9% [29/994], p=0.014). Details on concordance between tests are presented in Supplementary Table 2. Despite this association at an individual level, there was no statistically significant association between village-level seroprevalence and nPCR parasite prevalence for *P. falciparum* (p=0.3559) or *P. vivax* (p=0.1443).

**Table 1.** Characteristics of study participants and malaria indicators per village

Village	Age (years), median (IQR)	Female, % (n/N)	Previous malaria,^a^ % (n/N)	Microscopy			nPCR				Serology	
				*P. falciparum*, % (n/N)	*P. vivax*, % (n/N)	Total, % (n/N)	*P. falciparum*, % (n/N)	*P. vivax*, % (n/N)	Mixed species, % (n/N)	Total, % (n/N)	Pf-AMA, % (n/N)	Pv-AMA, % (n/N)
1	9 (6–15)	57.7 (49/85)	3.6 (3/84)	0 (0/85)	0 (0/85)	0 (0/85)	7.1 (6/85)	4.7 (4/85)	0 (0/85)	11.8 (10/85)	3.5 (3/3/85)	4.7 (4/85)
2	12 (5–35)	54.7 (52/95)	7.4 (7/95)	5.3 (5/95)	0 (0/95)	5.3 (5/95)	19.1 (18/94)	3.2 (3/94)	2.1 (2/94)	24.5 (23/94)	22.1 (19/86)	3.5 (3/86)
3	30 (20–45)	17.8 (13/73)	26.0 (19/73)	0 (0/73)	1.4 (1/73)	1.4 (1/73)	5.5 (4/73)	4.1 (3/73)	1.4 (1/73)	11.0 (8/73)	38.4 (28/73)	4.1 (3/73)
4	12 (9–15)	45.3 (58/128)	16.4 (21/128)	2.3 (3/128)	0.8 (1/128)	3.1 (4/128)	3.1 (4/128)	0 (0/128)	0 (0/128)	3.1 (4/128)	3.1 (4/128)	1.6 (2/128)
5	7 (5–10)	52.0 (53/102)	31.4 (32/102)	0 (0/102)	0 (0/102)	0 (0/102)	1.0 (1/102)	1.0 (1/102)	1.0 (1/102)	3.0 (3/102)	2.0 (2/102)	2.0 (2/102)
6	11 (6–35)	52.2 (47/90)	37.8 (34/90)	4.9 (4/81)	1.2 (1/81)	6.2 (5/81)	9.0 (8/89)	6.7 (6/89)	1.1 (1/89)	16.9 (15/89)	14.4 (13/90)	15.6 (14/90)
7	17 (9–30)	72.9 (51/70)	18.6 (13/70)	1.4 (1/70)	1.4 (1/70)	2.8 (2/70)	8.6 (6/70)	5.7 (4/70)	0 (0/70)	14.3 (10/70	4.3 (3/70)	17.4 (12/69)
8	12 (7–27)	48.9 (39/80)	11.3 (9/80)	0 (0/80)	0 (0/80)	0 (0/80)	0 (0/80)	1.3 (1/80)	1.3 (1/80)	2.6 (2/80)	6.3 (5/80)	38.8 (31/80)
9	15 (7–25)	41.4 (29/70)	40.4 (23/57)	0 (0/70)	0 (0/70)	0 (0/70)	1.4 (1/70)	1.4 (1/70)	0 (0/70)	2.8 (2/70)	8.6 (6/70)	75.7 (53/70)
10	16 (9–35)	60.2 (56/93)	44.1 (41/93)	1.1 (1/93)	1.1 (1/93)	2.2 (2/93)	2.2 (2/93)	6.5 (6/93)	1.1 (1/93)	9.7 (9/93)	18.3 (17/93)	23.7 (22/93)
11	10 (6–15)	56.1 (37/66)	40.9 (27/66)	0 (0/66)	0 (0/66)	0 (0/66)	0 (0/66)	3.0 (2/66)	0 (0/66)	3.0 (2/66)	13.6 (9/66)	0 (0/66)
12	16 (10–30)	45.5 (40/88)	39.8 (35/88)	0 (0/88)	0 (0/88)	0 (0/88)	0 (0/88)	1.1 (1/88)	0 (0/88)	1.1 (1/88)	14.8 (13/88)	0 (0/46)
13	4.5 (3–9)	71.7 (33/46)	21.7 (10/46)	0 (0/46)	0 (0/46)	0 (0/46)	0 (0/46)	4.4 (2/46)	0 (0/46)	4.4 (2/46)	0 (0/46)	0 (0/46)
14	10 (4–30)	67.2 (39/58)	24.1 (14/58)	0 (0/58)	0 (0/58)	0 (0/58)	1.7 (1/58)	0 (0/58)	0 (0/58)	1.7 (1/58)	5.2 (3/58)	1.7 (1/58)
Total	12 (7–30)	52.1 (596/1144)	25.5 (288/1130)	1.2 (14/1135)	0.4 (5/1135)	1.7 (19/1135)	4.5 (51/1143)	3.0 (34/1143)	0.6 (7/1143)	8.0 (92/1142)	11.0 (125/1135)	13.0 (147/1134)

^a^Previous malaria refers to self-reported history of malaria in the last year that was captured with a questionnaire.

### Seroconversion rates and reverse cumulative distribution

When samples from all villages were combined, the likelihood of individuals testing seropositive increased with age for both Pf-AMA (odds ratio [OR] 1.07 [95% CI 1.05 to 1.09], p<0.001) and Pv-AMA (OR 1.02 [95% CI 1.01 to 1.04], p<0.001), which was also apparent from the age-seroprevalence curves (Figure 1C, D). Profile likelihood analysis showed evidence for a change in transmission occurring approximately 15.5 y (p<0.001) and 11.5 y (p=0.010) prior to the survey for Pf-AMA and Pv-AMA, respectively. To examine age patterns in antibody responses in more detail, reverse cumulative distribution (RCD) plots were generated (Figure 1E, F). Higher antibody titres were detected in adults than younger ages. The difference in antibody levels for age groups (adults >15 y of age and children <15 y of age) was significant for both parasite species (p<0.001). When examining antibody responses in more detail in children, differences were observed between all age groups except between those <5 y of age and those 5–10 y of age. Of the five children <5 y of age who were Pf-AMA seropositive, *P. falciparum* infection was detected by nPCR in three of them, thus demonstrating current malaria exposure in this population. Among 14 children <5 y of age who tested positive for Pv-AMA, infection was detected by nPCR in only 4; the single 1-y-old child who was seropositive for Pv-AMA antibodies also tested positive for *P. vivax* infection using nPCR.

## Discussion

Following the epidemics in the early 2000s, a substantial reduction in malaria incidence was reported in Ethiopia.^17^ In 2005, Ethiopia introduced major scale-up efforts in malaria prevention and control, including the introduction of artemisinin-based combination therapy (ACT) as first-line treatment,^18^ large-scale distribution of long-lasting insecticidal nets, improved indoor residual spraying campaigns and training of 30 000 health extension workers in malaria diagnosis and treatment.^19^ This effort was followed by a decline in new malaria cases by 66% in 2011 as compared with the pre-intervention period.^1^ In line with this, the serological investigation in the present study revealed a significant decline in malaria transmission 15 y prior to the study for *P. falciparum* and 11.5 y for *P. vivax*. Higher antibody titres were detected in adults compared with children, in line with other studies and supporting the notion of antibody acquisition following repeated exposure to malaria infections. Serological responses to parasite antigens are informative tools to reflect the history of malaria and monitor malaria control programmes and have the potential to uncover heterogeneity in the effectiveness of malaria control interventions.^20,21^

The change time predicted for *P. falciparum* infections in the present study is earlier than the major scale-up of malaria interventions in the country. Of note, improved malaria control started before 2002 with the replacement of chloroquine with sulphadoxine–pyrimethamine as the first-line treatment for uncomplicated *P. falciparum* malaria around 1998 and improved access to insecticide-treated nets around the same time.^17^ In line with this, a nationwide decline in malaria incidence and death rates has been recorded since 2001.^17^ A similar rate of decline in the years prior to the massive scale-up was reported in a recent serological study in the northwestern part of the country that even showed an earlier predicted time point than the present study.^22^ The estimate of time since changes in malaria burden in the current study may thus be realistic. Moreover, rainfall and other climatic patterns may affect transmission regardless of government-imposed interventions.^23,24^ Variation in the change point estimate from seroconversion curves also depends on sample size and how sudden transmission changed.^25^

A strength of the current study is that it utilized both routine tests (microscopy) and improved tools (PCR and serology) to concurrently estimate current and historic levels of malaria transmission. Shortcomings include the limited number of examined villages and villagers and the lack of formal random selection in participant recruitment. The asymptomatic status of study participants give us confidence that no systematic bias was introduced. An additional limitation is the reliance on AMA-1 as the sole antigen. Although AMA-1 is a well-characterized marker of historical exposure at the population level,^26,27^ malaria exposure might have been missed by only measuring responses to a single antigen. Further information could be generated by measuring short-lived antibodies to indicate recent exposure to infection across all ages.^28^

Despite these shortcomings, the current findings support evidence for a decline in malaria burden. The current data further demonstrate that malaria transmission is still ongoing in the study area. While importation of malaria is possible, and an important source of infection in several low-endemic settings, our findings of detectable serological responses in children, often accompanied by PCR-detected infection, suggest that there is still non-negligible local transmission. Continued efforts are required to further reduce malaria in the region. Novel tools that can interrupt transmission and improved strategies on enhanced community case management to find the few remaining infections that plausibly maintain the infectious parasite reservoir are of paramount importance in accelerating progress towards elimination. The striking variation in malaria indicators between villages highlights the need for a better understanding of (variation in) the uptake of interventions and potentially tailor interventions to local needs to accelerate elimination efforts in the region.

## Supplementary Material

Supplementary Data

Supplementary Data
