# Supplementary material for: Serological evidence for a decline in malaria transmission following major scale-up of control efforts in a setting selected for Plasmodium vivax and Plasmodium falciparum malaria elimination in Babile district, Oromia, Ethiopia
Source: Trans R Soc Trop Med Hyg. 2019 Mar 30;113(6):305–11. doi: 10.1093/trstmh/trz005 (PMC6580689; doi:10.1093/trstmh/trz005)
Supplement: Supplementary Data [file trz005_supplementary_table_1.docx]

Supplementary table 1. Self-reported intervention utilization

| village | Roof type | | | Eave opening | LLIN ownership | LLIN utilization started | | | Spraying |
| --- | --- | --- | --- | --- | --- | --- | --- | --- | --- |
|  | Grass thatch | Wood and mud | Iron sheet |  |  | < 1 year | 1 - 5 years | 6 - 10 year |  |
| 1 | 24.7 (21/85) | 57.7 (49/85) | 16.5 (14/85) | 41.7 (35/84) | 70.6 (60/85) | 0 | 98.3 (59/60) | 1.7 (1/60) | 61.8 (47/76) |
| 2 | 93.7 (89/95) | 6.3 (6/95) | 0 (0/95) | 60.0 (57/95) | 35.8 (34/95) | 0 | 100.0 (34/34) | 0 | 70.5 (67/95) |
| 3 | 74.0 (54/73) | 26.0 (19/73) | 0 (0/73) | 61.6 (45/73) | 60.3 (44/73) | 0 | 100.0 (44/44) | 0 | 86.1 (62/72) |
| 4 | 3.9 (5/128) | 88.3 (113/128) | 7.8 (10/128) | 16.4 (21/128) | 78.1 (100/128) | 0 | 100.0 (100/100) | 0 | 94.5 (121/128) |
| 5 | 68.6 (70/102) | 31.4 (32/102) | 0 (0/102) | 66.7 (68/102) | 50.0 (51/102) | 0 | 100.0 (51/51) | 0 | 86.3 (88/102) |
| 6 | 14.4 (13/90) | 85.6 (77/90) | 0 (0/90) | 31.1 (28/90) | 64.4 (58/90) | 0 | 100.0 (58/58) | 0 | 98.9 (89/90) |
| 7 | 12.9 (9/70) | 85.7 (60/70) | 1.4 (1/70) | 57.1 (40/70) | 84.3 (59/70) | 1.7 (1/59) | 98.3 (58/59) | 0 | 98.6 (69/70) |
| 8 | 46.3 (37/80) | 37.5 (30/80) | 0 (0/80) | 43.8 (35/80) | 25.0 (20/80) | 5.0 (1/20) | 90.0 (18/20) | 0 | 63.3 (50/79) |
| 9 | 34.3 (24/70) | 57.1 (40/70) | 8.6 (6/70) | 77.6 (52/67) | 74.3 (52/70) | 17.3 (9/52) | 78.9 (41/52) | 3.9 (2/52) | 50.7 (35/69) |
| 10 | 21.5 (20/93) | 78.5 (73/93) | 0 (0/93) | 50.5 (47/93) | 88.2 (82/93) | 0 | 52.4 (43/82) | 47.6 (39/82) | 100.0 (93/93) |
| 11 | 1.5 (1/66) | 98.5 (65/66) | 0 (0/66) | 43.9 (29/66) | 87.9 (58/66) | 3.5 (2/58) | 32.8 (19/58) | 63.8 (37/58) | 98.5 (65/66) |
| 12 | 6.8 (6/88) | 93.2 (82/88) | 0 (0/88) | 38.6 (24/88) | 92.1 (81/88) | 0 | 33.3 (27/81) | 66.7 (54/81) | 67.1 (59/88) |
| 13 | 4.4 (2/46) | 58.7 (27/46) | 32.6 (15/46) | 23.9 (11/46) | 93.5 (43/46) | 0 | 67.4 (29/43) | 32.6 (14/43) | 91.3 (42/46) |
| 14 | 1.7 (1/58) | 51.7 (30/58) | 46.6 (27/58) | 51.7 (30/58) | 81.0 (47/58) | 0 | 48.9 (23/47) | 51.1 (24/47) | 75.9 (44/58) |
| Total | 30.8 (352/1144) | 61.5 (703/1144) | 6.4 (73/1144) | 46.7 (532/1140) | 69.0 (789/1144) | 1.7 (13789) | 76.6 (604/789) | 21.7 (171/789) | 82.2 (931/1132) |

LLIN, long lasting insecticidal nets.
