# Supplementary material for: Serological evidence for a decline in malaria transmission following major scale-up of control efforts in a setting selected for Plasmodium vivax and Plasmodium falciparum malaria elimination in Babile district, Oromia, Ethiopia
Source: Trans R Soc Trop Med Hyg. 2019 Mar 30;113(6):305–11. doi: 10.1093/trstmh/trz005 (PMC6580689; doi:10.1093/trstmh/trz005)
Supplement: Supplementary Data [file trz005_supplementary_table_2.docx]

Supplementary table 2. Concordance between tests

| Microscopy | | **PCR (Gold standard)** | | | **ROC test** | | | **Kappa test** | |
| --- | --- | --- | --- | --- | --- | --- | --- | --- | --- |
|  |  | Negative | *P. falciparum* | *P. vivax* | Sensitivity (%) | Specificity (%) | Accuracy (%) | Agreement | K (P-value) |
| *P. falciparum* | Positive | 0 | 15 | 1 | 28.1 (17.0-42.0) | 100 (96.4-100) | 96.4 | 96.40% | 0.43  (<0.0001) |
|  | Negative | 1084 | 40 | NA |  |  |  |  |  |
| *P. vivax* | Positive | 0 | 3 | 1 | 4.9  (0.5-16.5) | 99.7 (99.2-99.9) | 96.3 | 96.30% | 0.08  (<0.0001) |
|  | Negative | 1085 | NA | 39 |  |  |  |  |  |
| Serology | |  | | |  | | | Area under curve | |
| *Pf*AMA | Negative | 980 | 36 | NA | 73.7 | 92.1 | 91.2 | 0.83 (0.81-0.85) | |
|  | Positive | 106 | 22 |  |  |  |  |  |  |
| *Pv*AMA | Negative | 965 | NA | 29 | 70.7 | 98.1 | 88.5 | 0.799 (0.73 - 0.87) | |
|  | Positive | 137 |  | 12 |  |  |  |  |  |

PCR, polymerase chain reaction; NA, not available; ROC, receiver operating characteristics.
